# Fortification of Sago Starch With Egg Powder: Impacts on Starch and Noodle Properties

**DOI:** 10.1155/tswj/3631288

**Published:** 2026-07-20

**Authors:** Agus Budiyanto, Risfaheri Risfaheri, Christina Winarti, Widaningrum Widaningrum, Sari Intan Kailaku, Anna Sulistyaningrum, Irpan Badrul Jamal, Tatang Hidayat, Agung Hendriadi, Abdullah Bin Arif

**Affiliations:** ^1^ Research Center for Equipment Manufacturing Technology, National Research and Innovation Agency, Indonesia, brin.go.id; ^2^ Research Center for Process Technology, National Research and Innovation Agency, Indonesia, brin.go.id; ^3^ Research Center for Food Technology and Processing, National Research and Innovation Agency, Indonesia, brin.go.id; ^4^ Research Center for Horticulture, National Research and Innovation Agency, Indonesia, brin.go.id; ^5^ Research Center for Behavioral and Circular Economics, National Research and Innovation Agency, Indonesia, brin.go.id

**Keywords:** egg powder, fortification, noodles, sago, starch

## Abstract

Sago starch can potentially serve as a carbohydrate‐rich staple food substitute for rice in Indonesia. However, its limitations include low protein content and a lack of other essential nutrients. This study is aimed at enhancing sago starch′s nutritional value and functional properties to make it comparable to rice by fortifying it with whole egg powder and egg white powder. The fortified sago starch was evaluated for its nutritional value, pasting properties, and its application in noodle production. The results indicate that fortifying sago starch with either whole egg powder or egg white powder can increase its nutritional value and improve the quality of both the sago starch and the resulting noodles. Specifically, formulations containing 80% sago starch and 20% whole egg powder yielded a protein content of 8.20%. In contrast, those with 90% sago starch and 10% egg white powder achieved a protein content of 8.64%. The addition of whole egg powder or egg white powder changed how sago starch pastes, which changed how it gelatinizes and forms gels. Compared to pure sago starch, fortification caused a drop in peak viscosity (PV), trough viscosity (TV), and final viscosity (FV). The drop in breakdown viscosity (BV) shows that the structure was stronger when heated. Adding both types of egg powder made the surface of the noodles thicker. Egg white powder had the most noticeable positive effect on the surface of the noodles, making them more compact and less porous and cracked. This improvement makes the noodles last longer, taste better, and have a better texture. The results of this study provide important information for making sago‐based foods better and for using sago starch in more ways in the food industry.

## 1. Introduction

The pith of the sago tree (*Metroxylon sago*) is used to make sago starch. Before rice became widely used, it was a staple food in eastern Indonesia, especially in Papua, Maluku, and Sulawesi. Indonesia has significant potential for sago farming; it has approximately 1.25 million hectares of sago forests capable of producing 37.5 million tons of sago starch annually. This production originates from both natural sago woods and partially cultivated plantations [1]. The primary regions for sago cultivation in Indonesia are eastern Indonesia (including Papua, Sulawesi, and Maluku) and western Indonesia (specifically eastern Sumatra) [2]. As a forest plant that thrives in swampy areas, sago is a vital source of carbohydrates and has great potential as a substitute for rice, Indonesia′s primary staple food.

Sago starch is commonly consumed in traditional dishes, such as papeda, a type of porridge, along with various processed variations. Although sago starch is a potential source of carbohydrates and can serve as a substitute for rice in Indonesia, it has some significant drawbacks. Notably, it has a low protein content and lacks other essential nutrients. In 100 grams of dry sago starch, the nutritional breakdown shows 85.6 grams of carbohydrates, 0.6 grams of protein, 1.1 grams of fat, and 0.3 grams of fiber [3]. In comparison, 100 grams of rice contain 79 grams of carbohydrates, 7.13 grams of protein, 1.7 grams of fat, 0.12 grams of fiber, and various essential minerals beneficial to the body. Additionally, rice is a source of various B vitamins [4]. Given these differences, replacing rice with sago starch as a staple food could decrease protein and other important nutrients typically provided by rice. Therefore, enriching sago starch with protein and other nutrients is crucial to be adopted as a staple food.

Sago starch nutrient enrichment is not only intended to increase the protein content of sago starch to a minimum equivalent to the protein content of rice but also to improve the properties of sago starch so that it can make the properties of sago starch better. The ability to form dough in starch is influenced by the interaction of protein with water, which is an important functional property in influencing the properties of dough formation [5]. The lowest protein content of sago starch causes sago starch to have a very low water absorption capacity. Several research studies have shown that adding protein to sago starch can enhance its functional properties.

Sago starch contains 20%–30% amylose and 70%–80% amylopectin [6]. The ratio of amylose and amylopectin content will affect the properties of starch. The higher the amylose content, the starch is less dry, less sticky, and hygroscopic. Sago starch has elliptical granules, and its gelatinization temperature ranges from 60%–72°C, and the granule size is 20–60 *μ*m [7, 8]. Meanwhile, according to Maaruf et al. [9], the gelatinization temperature of starch is around 63°C–80°C.

One potential natural protein source to enrich sago starch is egg powder. Egg powder comes from chicken eggs that have been dried and ground into powder. Every 100 grams of chicken eggs contains essential nutrients, including 12.8 grams of protein, 11.8 grams of fat, 327 IU of vitamin A, and 256 milligrams of minerals. Eggs are renowned for their high‐quality protein, which is attributed to their complete composition of essential amino acids (EAAs) and high biological value, specifically 100% [10]. Whole egg powder typically contains around 37%–42% protein [11], whereas egg white powder contains between 74.5% and 79% protein [12]. The fat content in whole egg powder is approximately 40%, whereas egg white powder contains less than 1%. The high protein content in egg powder presents significant potential for use as a fortifier in sago starch enrichment. Additionally, egg powder is also rich in calcium (Ca) and magnesium (Mg). Egg protein has excellent functional properties in addition to its nutritional benefits. These include the ability to hold water, mix things together, and make gels, all of which are important for improving hydration, dough development, and the texture of starch‐based foods. Egg powder is easy to digest and has a neutral taste, which makes it easy to add to sago starch without changing the taste or smell of the sago. This is different from plant‐based proteins, which often need to be processed more to get rid of bad smells or antinutritional factors. Additionally, unlike dairy proteins, egg powder does not cause problems for people who are sensitive to lactose and is more stable when starch gelatinizes. Indonesia has a lot of chicken eggs, and the technology for processing eggs is both advanced and cheap. This makes it more practical and sustainable to use egg powder as a natural protein booster for sago starch.

The first thing to consider when selecting a natural fortifier to enhance the protein content of sago starch is how well it interacts with other ingredients and how easily it can be sourced. Egg powder has considerable potential as a natural fortifier for sago starch, given the abundance of chicken eggs in Indonesia. Therefore, this study is aimed at enhancing the nutritional value and functional properties of sago starch, making it comparable to rice through the incorporation of whole egg powder and egg white powder.

## 2. Materials and Method

### 2.1. Materials and Characterization

**Figure 1:**
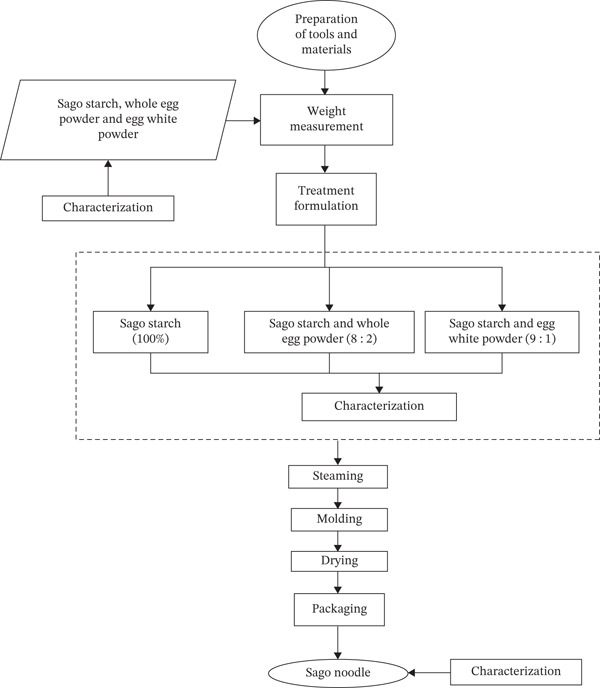
Fortified noodle production methodology.

The research materials used in this study included sago starch sourced from Papua and Riau, whole egg powder, egg white powder, and various chemicals for analysis. The equipment comprised a scale, pH meter, Brookfield viscometer, blender, stopwatch, SEM [Scanning Electron Microscopy] Hitachi SU3500, atomic absorption spectroscopy (AAS), texture analyzer (Taxt‐Plus), and various glassware. The activities carried out were the characterization of raw materials (sago starch, whole egg powder, and egg white powder), characterization of fortification results, and characterization of sago noodles (Figure 1). The characterization of raw materials and fortified products included proximate analysis, color (L^∗^, a^∗^, b^∗^, C^∗^[chroma], and Hue [hue angle]), starch, amylose, amylopectin, dietary fiber, and mineral content. At the same time, the characterization of the fortified results includes proximate analysis, color (L^∗^, a^∗^, b^∗^, C^∗^, Hue), starch, amylopectin, amylose, dietary fiber, mineral content, EAAs, and pasting properties. Characterization of sago noodles includes proximate analysis, EAAs, minerals, texture profile, color, and SEM.

### 2.2. Proximate Analysis

Proximate analysis includes moisture content, protein, ash content, and carbohydrates. The levels of moisture, protein, lipid, and ash in starches were determined following the official AOAC methods [13].

### 2.3. Starch Content Analysis

Starch content measurements were performed on sago starch and fortified ingredients. The total starch content comprises both amylopectin and amylose. Amylopectin content was determined by measuring the total starch content following ISO 5377, whereas amylose content was assessed based on the method described by Hoover and Ratnayake [14].

### 2.4. Mineral Analysis

The mineral content in sago starch raw materials and fortified ingredients, including Ca, Mg, potassium (K), sodium (Na), and iron (Fe), was analyzed to determine their nutritional composition and potential fortification benefits. The analysis was conducted using spectrometry, following the method described by Poitevin [15], to ensure accurate quantification of each mineral component.

### 2.5. Amino Acid (AA)Analysis

AA analysis was performed on sago starch and fortified flour, including the quantification of arginine, threonine, histidine, isoleucine, leucine, lysine, methionine, phenylalanine, and valine [16].

### 2.6. Color Measurements

A Chroma Meter (Konica Minolta CR‐400, Japan) with a D65 illuminant was used to look at the starch and noodle materials. The CIE L^∗^, a^∗^, and b^∗^ color space was used to do the assessment. The L∗ value ranges from 0 (black) to 100 (white) and shows how light or dark a color is. The a∗ value shows the red–green spectrum, with negative values showing green and positive values showing red. The b∗ value shows the blue–yellow spectrum. Negative values mean blue, and positive values mean yellow. The a∗ and b∗ values were used to figure out the C∗, which shows how bright or saturated a color is. Higher C∗ values mean colors are more vibrant or saturated, whereas lower C∗ values mean colors are less vibrant or muted. The Hue is the main color that is seen, and it is found by using the arctangent connection between the a∗ and b∗ values. The Hue is given in degrees: 0° is the positive a∗ axis (red), 90° is the positive b∗ axis (yellow), 180° is the negative a∗ axis (green), and 270° is the negative b∗ axis (blue).

### 2.7. Pasting Properties Analysis

Viscosity was measured using a Rapid Visco Analyzer (RVA4500, Perten, Sweden) under controlled conditions to ensure accuracy and consistency. The instrument settings were as follows: an initial temperature of 50°C, held for 1 min, followed by heating to 95°C over 3 min 42 s, then maintained at 95°C for 2 min 30 s. The sample was subsequently cooled to 50°C over 3 min 48 s and held at 50°C for a final 2 min. The STD1 profile was used in this test, based on AACC method 76–21.01, ICC standard no. 162, as briefly described in the previous research [17].

### 2.8. Texture Properties Analysis

The textural properties of cooked noodles were analyzed using a Taxt‐Plus texture analyzer (Stable Micro Systems, London, England). The noodles were first cooked according to standard methods, and the measurements were taken at room temperature for 10 min postcooking. Hardness, adhesiveness, springiness, cohesiveness, gumminess, chewiness, and resilience were analyzed using an HDP/PFS probe (a rectangular probe 50 mm long and 38 mm wide) and calculated through texture profile analysis (TPA). For testing, three strands of cooked noodles were placed parallel on the platform, with determinations made under the following conditions: strain set to 75%, pretest, test, and posttest speeds at 0.8 mm/s, and an interval time of 2 s [18].

### 2.9. SEM

The starch and noodle samples were the first step fixated in 2.5% glutaraldehyde for 2 h. Following the fixation, the samples were rinsed four times with cold phosphate buffer (0.1 M at 4°C). A secondary fixation step was using 1% osmium tetroxide for 1.5 h. The samples were then dehydrated through a graded ethanol series (30%, 50%, 70%, 90%, and 100%), with each step lasting 5 min. Isoamyl acetate was then applied to replace any residual ethanol. After critical point drying, surface and cross‐sectional images of the noodle samples were scanned using a SEM (Quanta‐200; FEI Ltd., Eindhoven, Netherlands) at an accelerating voltage of 1.0 kV. The micrographs were obtained at a magnification of 120x [18].

### 2.10. Statistical Analysis

Data were analyzed with a one‐way analysis of variance. The significant differences among the treatment means were determined by least significant difference (LSD) at a probability level of 5%. Statistical analyses of data were performed by SAS Portable Version 9.1.3. The data were reported as mean ± standard deviation (SE) of five replications.

## 3. Results and Discussions

### 3.1. Nutritional Content of Sago Starch, Whole, and White Egg Powder

Sago starch is rich in carbohydrates but lacks protein and other essential nutrients. In contrast, rice not only contains high levels of carbohydrates but also provides superior amounts of protein and other nutritional content compared to sago starch (Table 1). If sago starch were to replace rice as a staple food, the consumption of protein and other nutrients would decrease unless additional nutrients from other foods are added. The average rice consumption in Indonesia is 220 grams per person per day [20]. Based on the protein content data for rice and sago starch (Table 1), this would result in a potential loss of approximately 14.67 grams of protein per person per day, assuming the same consumption level, if the staple food switched from rice to sago.

**Table 1:** Nutritional content of sago starch, rice, and egg powder.

Parameters	Rice^a^	Sago starch	Whole egg powder	White egg powder
Moisture (%)	11.62	8.87 ± 0.32	0.95 ± 0.03	5.18 ± 1.29
Ash (%)	0.8	0.10 ± 0.02	3.58 ± 0.54	4.45 ± 0.98
Fat (%)	0.66	0.35 ± 0.10	56.88 ± 2.33	0.15 ± 0.02
Protein (%)	7.13	0.46 ± 0.10	33.69 ± 1.43	82.35 ± 2.34
Carbohydrates (%)	79	90.23 ± 0.24	4.89 ± 1.12	7.87 ± 1.43
Dietary fiber (%)	0.12	3.48 ± 0.15	2.57 ± 0.56	1.21 ± 0.63
Mineral				
Ca (mg/100 g)	28	15.03 ± 0.05	61.26 ± 1.23	7.21 ± 1.76
Mg (mg/100 g)	25	42.47 ± 0.87	74.84 ± 4.32	11.00 ± 1.67
K (mg/100 g)	115	nd	0.77 ± 0.04	163.00 ± 3.45
Na (mg/100 g)	27	nd	1.71 ± 0.65	403.00 ± 5.47
Fe (mg/100 g)	0.8	2.00 ± 0.02	0.16 ± 0.03	0.10 ± 0.01

Abbreviation: nd, not detected.

^a^ Source: Wijaya et al. [19].

The results of research by Syartiwidia et al. [21] in an area that consumes sago as a staple food, namely Meranti, Riau Province, Indonesia, the average level of protein intake is classified as very low, accounting for only 38.3% of the daily protein requirement. These data indicate that individuals who consume sago starch as a staple food are unable to meet their protein needs, as sago contains minimal protein. Therefore, sago consumption must be added protein from other food sources. Fortifying sago starch with egg powder can increase its protein content and nutritional value. In addition to being rich in protein, egg powder is an excellent source of minerals such as Fe, Ca, phosphorus, and K (Table 1). Furthermore, it is high in vitamins, including vitamin E, vitamin A, vitamin B2, folic acid, and vitamin B12 [22].

Whole egg powder exhibited the highest Ca and Mg contents compared to egg white powder and sago flour, reaching 61.26 and 74.84 mg/100 g, respectively. This is attributed to the accumulation of Ca and Mg primarily in egg yolk granules, resulting in higher concentrations in whole egg powder. Hühnereigelbfraktionen et al. [23] reported that egg yolk contains Ca, Mg, copper, Fe, and zinc, with nearly all of these minerals, except copper being concentrated in yolk granules. In contrast, egg white powder was rich in K and Na, with concentrations of 163 and 403 mg/100 g, respectively, whereas these minerals were present at very low levels or were undetectable in sago flour. Na is dissolved in the albumen to maintain osmotic pressure, explaining its higher concentration in egg white; accordingly, K and Na are the major minerals in albumen [24].

The protein content of sago starch (original) from the research results was 0.46*%* ± 0.10*%*, equivalent to the results of Nurmiati et al. [25] was 0.57*%* ± 0.01*%*. Enrichment of sago starch by adding whole egg powder with a ratio of 80% sago starch and 20% whole egg powder succeeded in increasing the protein content to 8.2 ± 1.20 and at a mixing ratio of 90% sago starch and 10% egg white powder succeeded in enhancing the protein content to 8.64*%* ± 2.44*%* (Table 2). Eggs are animal proteins known for their high protein and AA quality, better than vegetable protein sources. Substitution of sago flour with whole egg powder (8:1) resulted in higher Ca and Mg contents than substitution with egg white powder (9:1), reaching 50.56 and 39.32 mg/100 g, respectively. Conversely, substitution with egg white powder produced higher Na and K contents, at 40.31 and 16.31 mg/100 g. These findings are consistent with the mineral characteristics of whole egg and egg white powders [23, 24]. Eggs are a valuable source of essential minerals such as Fe, Ca, phosphorus, and K, as well as vitamins A, E, B2, folate, and vitamin B12 [22, 26]. Although the Fe content of the substituted flours (1.54%–1.81%) was lower than that of sago flour, both substitution treatments still provided significant nutritional benefits. In contrast, sago flour is characterized by relatively low mineral content and is poor in protein and fat [27]. This fortified sago starch has nutritional content equivalent to rice, so it can be used as a staple food to replace rice (Tables 1 and 2).

**Table 2:** Nutritional content of sago starch after fortification with egg powder.

Parameters	80% sago starch and 20% whole egg powder	90% sago starch and 10% egg white powder
Moisture (%)	7.29 ± 0.98^a^	8.50 ± 1.01^a^
Ash (%)	0.71 ± 0.02^a^	0.53 ± 0.02^b^
Fat (%)	0.30 ± 0.01^a^	0.33 ± 0.01^a^
Protein (%)	8.20 ± 1.20^a^	8.64 ± 2.44^a^
Carbohydrates (%)	83.51 ± 2.36^a^	81.99 ± 3.89^a^
Dietary fiber (%)	3.25 ± 0.87^a^	3.25 ± 0.93^a^
Mineral		
Ca (mg/100 g)	26.59 ± 2.76^a^	14.25 ± 1.29^b^
Mg (mg/100 g)	50.56 ± 4.65^a^	39.32 ± 3.27^b^
K (mg/100 g)	0.19 ± 0.01^b^	16.31 ± 2.21^a^
Na (mg/100 g)	0.43 ± 0.02^b^	40.31 ± 4.49^a^
Fe (mg/100 g)	1.54 ± 0.77^a^	1.81 ± 0.07^a^

*Note:* Values are means ± standard error, means followed by different letters in the same lines indicate the significant difference by the LSD test.

### 3.2. Sago Starch Properties

The addition of egg products (whole egg powder or egg white powder) into sago starch significantly increased the protein content, and it simultaneously decreased the percentage of total starch, amylose, and amylopectin fraction due to the dilution of the original starch components of sago. The addition of egg powder (containing egg yolk) to the 80/20 formulation affected the color, namely decreasing the brightness (L∗), increasing the intensity of the yellow color (b∗ and C∗ values), and shifting the hue towards yellow. The formulation of 80% sago starch +20% whole egg powder had a darker color (lower L∗ value) and an increasing yellow intensity (higher b∗ and C∗ values and a Hue shift towards yellow). It was due to the presence of yellow pigment in the egg yolk. Meanwhile, the formulation of 90% sago starch +10% egg white powder showed color properties almost identical to pure sago starch because egg white powder did not contribute pigment (Table 3).

**Table 3:** Properties of sago starch.

Parameters	Sago starch (100%)	80% sago starch and 20% egg powder	90% sago starch and 10% egg white powder
Protein (%)	0.46 ± 0.10^b^	8.20 ± 1.20^a^	8.64 ± 2.44^a^
Starch (%)	84.74 ± 0.44 ^a^	64.75 ± 0.20^c^	76.51 ± 4.29^b^
Amylose (%)	32.44 ± 0.53 ^a^	24.53 ± 0.41^b^	29.26 ± 2.78^a^
Amylopectin (%)	52.30 ± 0.96^a^	40.23 ± 0.61^b^	47.24 ± 3.62^a^
Color			
L∗	94.62 ± 2.45^a^	73.45 ± 2.87^b^	94.72 ± 5.43^a^
a∗	6.13 ± 1.12^a^	3.12 ± 0.89^b^	6.16 ± 1.02^a^
b∗	2.35 ± 0.54^b^	15.67 ± 2.79^a^	3.67 ± 0.67^b^
C∗	6.57 ± 1.01^b^	14.35 ± 2.45^a^	7.13 ± 1.47^b^
Hue	20.96 ± 1.22^b^	79.89 ± 4.49^a^	83.65 ± 4.47^a^

*Note:* Values are means ± standard error, means followed by different letters in the same lines indicate the significant difference by the LSD test.

According to Hayuningtyas et al. [12], the drying process in egg powder production reduces the water content to less than 10% and causes a color change due to the Maillard reaction, which leads to the formation of melanoidin compounds. Egg white contains glucose as a reducing sugar and protein, then reacts to form a brownish‐yellow compound.

In its native form, sago starch has limited functionality due to its compact constituent structure, which results in low solubility of the starch granules, reduced gel clarity, a shorter shelf life, and limited emulsifying capability [28]. Therefore, chemical and physical modifications of sago starch have been widely studied to obtain the desired physicochemical and rheological properties [29].

Lecithin contained in egg yolk functions as an emulsifier and product stabilizer [30]. Egg yolk protein forms a barrier that protects oil droplets, thereby improving the surface properties of the emulsion and producing the desired texture (improving the dough structure) in the product [31]. According to Jading et al. [32], sago starch has a very low protein content (0.46%), resulting in a very low water absorption capacity. Therefore, several researchers have studied the enrichment of protein content in starch or powder to improve its functional properties. Otegbayo et al. [33] included soy powder in tapioca, whereas Akinwale et al. [34] added soy protein isolate (SPI) to cassava starch‐based pudding powder. The interaction of protein and water is closely related to the AA composition, protein conformation, and hydrophilic/hydrophobic balance, significantly affecting the water binding capacity.

The pasting qualities are crucial for forecasting the pasting behavior and capacity of the powder samples. Pasting features represent one of the most frequently evaluated quality criteria, possibly due to the well‐established methodologies that reliably predict powder or starch quality. The pasting properties are shown in Table 4.

**Table 4:** Pasting properties of the sago starch composite.

Parameters	Formulation		
	100% sago starch	80% sago starch and 20% egg powder	90% sago starch and 10% egg white powder
Peak viscosity (PV, cP)	4857.00 ± 222.76^a^	3396.00 ± 155.76^b^	3917.00 ± 179.65^b^
Trough viscosity (TV, cP)	1610.00 ± 38.44^a^	1303.00 ± 31.11^c^	1481.00 ± 35.36^b^
Breakdown viscosity (BV, cP)	3247.00 ± 192.91^a^	2093.00 ± 124.35^b^	2436.00 ± 144.73^b^
Final viscosity (FV, cP)	2649.00 ± 84.27^a^	1910.00 ± 60.76^c^	2271.00 ± 72.24^b^
Setback viscosity (SV, cP)	1039.00 ± 48.53^a^	607.00 ± 28.35^c^	791.00 ± 36.94^b^
Peak time (min)	3.87 ± 0.02 ^c^	4.07 ± 0.02^a^	3.93 ± 0.02^b^
Pasting temperature (°C)	77.40 ± 0.18^b^	78.30 ± 0.18^a^	78.25 ± 0.18^a^

*Note:* Values are means ± standard error, means followed by different letters in the same lines indicate the significant difference by the LSD test.

Enrichment of sago starch protein content by adding whole egg powder or egg white powder decreases the peak viscosity (PV), trough viscosity (TV), breakdown viscosity (BV), final viscosity (FV), and setback viscosity (SV) values compared to pure sago starch. It is mainly due to the influence of starch components and the possibility of interaction between proteins (from egg products) and sago starch molecules, which affect the swelling and stability of starch granules during heating. The PV value indicates the optimal swelling capacity of starch when heated. The decrease in the PV value in a mixture of 80% sago starch and 20% whole egg powder indicates that the addition of whole egg powder reduces the swelling ability of starch, whereas mixing 90% sago starch and 10% egg white powder has a more moderate effect on starch swelling.

The incorporation of protein components to sago starch diminishes PV, facilitating the attainment of composite dough viscosity during mixing or achieving peak viscosity at a moderate temperature, which is advantageous and preferable. The findings align with the study of Majzoobi et al. [35], which indicated that the incorporation of SPI into maize starch diminished all viscosities (peak, trough, breakdown, final, and setback) without influencing the pasting temperature.

Reduced PV may result from the lower starch content in composite sago starch. Peak viscosity indicates the maximum swelling of the flour before disintegration [36] and represents the viscosity load expected during mixing. The relatively low peak viscosity of composite powder suggests its suitability for products requiring minimal gel strength and high flexibility. Additionally, PV assesses the paste′s ability to withstand breakdown during the cooling period. The fluctuation in peak viscosity is frequently linked to the swelling capacity of starch and the rate of disrupted starch granules [37].

The TV or holding strength of composite flour represents the minimal viscosity after the peak, indicating that the starch granules remain undisturbed when the flour paste is subjected to a holding period at a constant temperature, duration, and shear stress [37]. The holding viscosity (trough) measures the viscosity of disturbed and swollen starch granules subjected to shear and heating. It indicates the susceptibility of different starches to disintegrate when hydrated, especially in starches with reduced amylose content. In the present study, the incorporation of whole egg powder to sago starch results in a decrease in viscosity stability during the heating and stirring process, whereas the incorporation of egg white powder to sago starch also reduces viscosity stability, though to a lesser extent than whole egg powder.

BV value is the difference between PV and TV, indicating the degree of collapse of the starch structure during the heating period. Lower BV values in formulations containing whole egg powder or egg white powder suggest a rise in structural stability during heating due to reduced damage to starch granules. BV is a numerical value obtained by subtracting the holding viscosity from the peak viscosity. The holding viscosity of starch reflects its ability to maintain stability at high temperatures, whereas BV indicates its tendency to retrograde [37, 38]. Deterioration viscosity serves as an indicator of paste stability [39]. It implies that a higher starch content results in a higher BV. Heating can disrupt the composite granules; therefore, when using this type of sago starch composite dough with egg shells, low heating must be applied to obtain the desired swelling.

The FV value represents the viscosity at the end of the cooling process and indicates the gel‐forming ability. The addition of whole egg flour or egg white flour to sago starch decreases the FV value, suggesting a reduction in gel‐forming ability or a modification of the gel structure. The SV value reflects the tendency of retrogradation or the rearrangement of starch molecules during cooling [36]. SV represents the pasting phase curve after the cooling of starch, during which reassociation, retrogradation, or rearrangement of starch molecules occurs. It is a numerical value obtained by subtracting the FV from the holding viscosity. Peroni et al. [40] demonstrated that powder with a low setback value may have low amylose content with high molecular weight. A lower tendency for retrogradation results in a lower setback value during the cooling of flour‐based products [41]. Conversely, high setback values are associated with syneresis.

There were significant differences in FV and setback among the powder samples. The composite powder could form a much better powder paste that could find application in the food industry. SV indicates gel stability and potential for retrogradation [42]. The viscosity changes while cooling were mainly due to amylose molecular reassociation, and low SV indicated a low rate of starch retrogradation.

A slight increase in the pasting temperature value in sago starch with whole egg flour and egg white flour suggests that protein components raise the temperature required to initiate starch gelatinization. The slight increase in peak time and pasting temperature indicates that the interaction between protein and starch helps form a more stable network, resulting in noodles with the desired texture. Peak time is the point when maximum viscosity is reached, measured in minutes [43]. The peak temperature, or initial gelatinization temperature, is the temperature at which starch granules begin to absorb water, as indicated by an increase in viscosity [42]. The results showed that the initial gelatinization temperature of sago starch composite flour with various protein source powders ranged from 77.40°C to 79.00°C. Sago starch, used as a control, had the lowest pasting temperature compared to composite flours, indicating that sago starch can absorb water earlier than the others. According to Li et al. [44], the initial gelatinization temperature is driven by heating medium conditions, starch granule size, and the fat and protein content of the starch. The higher the fat and protein content in the starch, the stronger the interaction between these components and the starch granules, which inhibits the release of amylose and requires more energy to facilitate its release. The composite flour used in this study contains protein as a complementary agent, which is highly likely to interact with the carbohydrates in sago starch. Consequently, this interaction may lead to a higher initial gelatinization temperature.

Overall, the enrichment with whole egg powder or egg white powder to sago starch altered the pasting properties of the starch, which in turn affected its gelatinization and gel formation behavior. Although there was a decrease in PV, TV, and FV values compared with pure sago starch, the lower BV values indicated an increase in structural stability during heating. It could have a positive impact on improving the consistency of noodle dough and texture characteristics after cooking.

**Figure 2:**
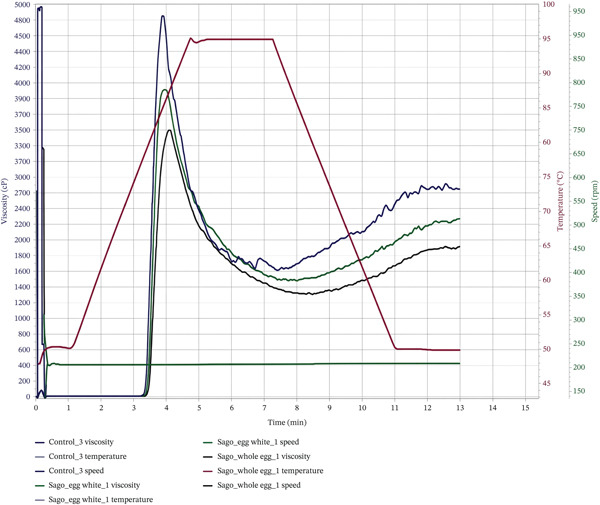
Pasting (gelatinization) profiles of sago starch, sago starch fortified with whole egg powder, and sago starch fortified with egg white powder.

Analysis of the pasting profile graph shows that sago starch (blue line) has the highest peak viscosity but also indicates a sharp decrease in viscosity, indicating that the starch structure is more easily damaged. Sago starch fortified with whole egg flour (black line) and egg white flour (green line) affects the functional properties of starch. The decrease in the viscosity peak indicates the interaction between protein and starch molecules, which inhibits maximum granule development (Figure 2).

Adding whole egg powder and egg white powder to sago starch increased the thermal and mechanical stability of starch, making it more suitable for products requiring high heat processing or long‐term storage. The decrease in porosity and cracks in the morphology (as mentioned in the previous narrative) may be related to presence of a protein‐starch network that can strengthen the structure.

Each treatment affected starch gelatinization differently. Sago starch showed high viscosity but was less stable, whereas sago starch fortified with whole egg flour and egg white flour was more stable to heating and stirring, although its peak viscosity was lower. This study suggests that fortification with egg flour can improve the functional quality of starch for application in food products, such as noodles.

### 3.3. Nutritional Content of Sago Noodle

Similar to the results of the nutritional analysis of sago starch and sago starch enriched with egg flour, the nutritional content of sago noodles enriched with egg flour also increased (Table 5). The most significant improvement was the rise in protein and AA content due to the fortification with egg powder. Protein quality is determined by the completeness of its AA profile. Insufficient intake of EAAs can interfere with metabolic processes, directly impacting children′s linear growth [45]. A deficiency in EAAs can hinder growth and lead to various health problems [46]. Protein intake is essential for strengthening and repairing damaged tissue cells. Furthermore, dietary protein is a determining factor in children′s brain development and growth.

**Table 5:** Nutrients composition of sago noodle.

Parameters	100% sago starch	80% sago starch and 20% whole egg powder	90% sago starch and 10% egg white powder
Proximate			
Moisture (%)	13.53 ± 3.21^a^	12.90 ± 1.11^a^	10.74 ± 1.27^a^
Ash (%)	0.06 ± 0.01^c^	0.71 ± 0.02^a^	0.37 ± 0.01^b^
Fat (%)	0.39 ± 0.02 ^b^	6.64 ± 1.06^a^	0.35 ± 0.01^b^
Protein (%)	0.46 ± 0.03^c^	10.57 ± 1.02 ^a^	8.13 ± 1.13^b^
Carbohydrate (%)	85.57 ± 2.31^a^	69.19 ± 2.37^b^	80.42 ± 3.39^a^
Nonessential AAs			
Aspartic acid	0.073 ± 0.002^c^	0.895 ± 0.021^a^	0.632 ± 0.027 ^b^
Glutamic acid	0.166 ± 0.011^b^	1.469 ± 0.143^a^	1.313 ± 0.211^a^
Serine	0.019 ± 0.001 ^b^	0.703 ± 0.099^a^	0.521 ± 0.097^a^
Glisin	0.010 ± 0.001^c^	0.523 ± 0.054^a^	0.267 ± 0.065 ^b^
Alanine	0.052 ± 0.002^c^	0.507 ± 0.053^a^	0.397 ± 0.031 ^b^
Proline	0.004 ± 0.000^c^	0.608 ± 0.065^a^	0.204 ± 0.009^b^
Tirosine	0.002 ± 0.000^c^	0.402 ± 0.021^a^	0.256 ± 0.022^b^
Cysteine	0.046 ± 0.002^c^	0.381 ± 0.007^a^	0.113 ± 0.003^b^
Total	0.372	5.488	3.70
Essential AAs			
Histidine	0.017 ± 0.001^c^	0.329 ± 0.036 ^a^	0.122 ± 0.013^b^
Arginine	0.021 ± 0.002^c^	0.873 ± 0.054^a^	0.405 ± 0.043^b^
Threonine	0.009 ± 0.000^c^	0.396 ± 0.041^a^	0.222 ± 0.027^b^
Valin	0.016 ± 0.001^c^	0.500 ± 0.005^b^	0.640 ± 0.044^a^
Methionine	0.009 ± 0.001^c^	0.130 ± 0.002^b^	0.382 ± 0.076^a^
Isoleucine	0.010 ± 0.001^c^	0.408 ± 0.012^b^	0.562 ± 0.032^a^
Leucin	0.010 ± 0.001 ^c^	1.054 ± 0.078^a^	0.760 ± 0.074^b^
Phenylalanine	0.011 ± 0.002^c^	0.715 ± 0.065^a^	0.574 ± 0.029^b^
Lysine	0.007 ± 0.001^c^	0.409 ± 0.071^a^	±0.037 ^a^
Total EAA	0.11	4.814	4.117
EAA/NEAA (%)	29.6	87.7	111.3
Mineral			
Ca (mg/100 g)	20.96 ± 3.32^c^	577.72 ± 21.22^a^	98.11 ± 3.44^b^
Mg (mg/100 g)	50.55 ± 2.32^c^	288.11 ± 16.78^a^	113.50 ± 10.21^b^
K (mg/100 g)	11.03 ± 1.04^b^	501.42 ± 28.98^a^	13.62 ± 1.63^b^
Na (mg/100 g)	9.00 ± 1.03^b^	492.99 ± 32.28^a^	7.95 ± 2.21^b^
Fe (mg/100 g)	2.92 ± 0.97^b^	4.31 ± 0.82^a^	6.10 ± 1.11^a^

*Note:* Values are means ± standard error, means followed by different letters in the same lines indicate the significant difference by the LSD test.

Enrichment of sago using egg powder will increase the protein as well as the AAs content of sago noodles. Eggs are well‐known animal proteins with high protein/AA quality that fulfill the human recommended daily allowance (RDA) better than vegetable protein sources, and EAAs deficiency can depress growth and lead to many health problems [46]. Protein and AAs are primary components of eggs. Protein and AAs and play critical roles as they present the main part of the muscle, body function, hormones, enzymes and body fluids [47, 48]. Among AAs in eggs, lysine was found to be 0.929, 1.182, and 0.76 g/100 g in fresh whole eggs, yolk, and albumen, respectively; for methionine, the corresponding values were 400, 375, and 396 [49]. The part of eggs added will influence the performance of enriched sago. According to Attia [50], among different parts of the whole edible parts of eggs (albumen + yolk), albumen, and yolk itself, the yolk had a greater (*p* ≤ 0.05) concentration of crude protein and AAs than the whole edible parts of eggs, which in turn had greater values (*p* ≤ 0.05) of protein than the albumen.

The mineral content of noodles (Table 5) produced by substituting sago flour with whole egg powder at a ratio of 8:2 was the highest compared to the other treatments. These noodles contained the greatest amounts of Ca, Mg, K, and Na and Fe. This was followed by noodles produced using the substitution of sago flour with egg white powder at a ratio of 9:1 exhibited the highest Fe content (6.10 mg/100 g). In contrast, noodles made from 100% sago flour showed the lowest mineral contents for Ca, Mg, K, Na, and Fe. Eggs are food materials with high biological value, providing proteins, AAs, vitamins, and essential minerals, including phosphorus, chlorine, K, Na, sulfur, Ca, Mg, and Fe [26]. The mineral content of noodles with 20% whole egg powder substitution showed the highest values, which can be attributed to the accumulation of minerals such as Ca and Mg in the yolk granules, whereas K and Na are distributed in both the egg white and yolk, with a tendency to be higher in the egg white. Egg yolk is a rich source of minerals, including Ca, Mg, Fe, and zinc, which are generally concentrated in the granular fraction [23]. Although egg white contains relatively higher levels of K and Na [24], the use of whole egg powder results in a greater overall mineral contribution due to the combination of both fractions as well as the higher substitution level (20%) compared to egg white powder (10%). According to Power et al. [51] and Cormick and Belizán [52], Ca is a highly important nutrient due to its role in reducing the risk of bone fractures, osteoporosis, and premenstrual syndrome, supporting bone health, and lowering the risk of hypertension by reducing blood pressure and cholesterol levels. Substitution of sago flour with whole egg powder or egg white powder represents a promising approach to producing noodles that are not only high in protein but also rich in essential minerals beneficial to health.

### 3.4. Non‐NEAAs

The increase in non‐EAA levels in sago with whole egg flour was higher than in egg white flour in all types of NEAAs (Table 5). The aspartate and glutamic shafts are the second most high types of NEAA. Asam aspartate increased to 0.895*%* ± 0.021*%* and 0.632*%* ± 0.027*%* and glutamic acid increase to 1.469*%* ± 0.143*%* and 1.313*%* ± 0.211*%*, respectively. According to Gunawan et al. [53], among all AAs in sago flour derived from South Sulawesi, Indonesia, the highest content were aspartate (1.846%) and glutamide acids (2.772%). Compared to the research, the sago flour from papua was comparable with these two compounds were also the highest, with slightly different amount of 0.073% and 0.166%. Glutamic acid and aspartic acids were the predominant NEAAs in the whole edible parts of eggs and the egg yolk; consequently, the increase of these two NEAAs in sago with whole eggs is higher than that of egg white. Among NEAAs, glutamic acid is the chief NEAA, and Serine is important in biosynthesis of purine and pyrimidines.

### 3.5. EAAs

The two highest types of AAs were leucine and isoleucine with scores of 1.054 and 0.760 and 0.409 and 0.450, respectively, for sago noodles with egg flour and egg white flour. Leucine, followed by lysine and arginine, were the most abundant EAAs in eggs. Moreover, the principal AAs in egg albumen were leucine, lysine, and valine, with the glutamic acid and aspartic acid were the chief NEAAs. Leucine is one of the most important types of EAAs because it helps protein synthesis. The high content of leucine, lysine and arginine in eggs albumin caused a drastic increase in sago flour enriched with egg flour and egg white flour. The leucine level increased most in sago flour after the addition of whole egg flour and egg white flour, which were from 0.010% to 1.054*%* ± 0.078*%* and 0.760*%* ± 0.074*%*, respectively. The second highest increase was Phenil alanine which increased from 0.11% to 0.715*%* ± 0.065*%* and 0.574*%* ± 0.029*%*. The third highest increased types of AAs was Isoleucine with scores increase from 0.01% to 0.406% and 0.562%, respectively, for sago noodles with egg flour and egg white flour. The addition of Sago starch with whole egg flour with a ratio of 8:2 has a higher AAs composition than the treatment of the ratio of sago and egg white (9:1) as seen in Table 5. The leucine content in egg flour is around 10.5 g/100 g. The RDA of leucine is 39 (2730 mg/day), lysine is 30 (2100 mg/day), and isoleucin is 20 (1400 mg/day) [50].

Phenylalanine—an EAAs for the biosynthesis of norepinephrine and epinephrine—and valine—which is important AAs for maintaining muscles, as well as for the regulation of the immune system—along with leucine and isoleucine, are branched‐chain AAs and represent about two‐thirds of AAs in the body protein [50]. In whole edible parts of eggs, branched‐chain AAs is amounted to ~45% of EAAs.

### 3.6. EAA/NEAA Ratio

The ratio of EAAs to non‐NEAAs is crucial for optimal protein synthesis and nutrient utilization, as the body needs a balanced supply of both types to build and repair tissues. Moreover, maintaining an appropriate EAA/NEAA ratio is essential for maximizing protein efficiency and overall health. The result research showed that EAA/NEAA ratio of sago was 29.6%, and increase to 87.7% in Sago + whole eggs and 111.3% in sago + white egg flour. According to Attia et al. [50] the EAAs/NEAAs, the was the highest in the albumen and lowest in the yolk. The values for the whole edible parts of the eggs were intermediate.

The importance of sago to be enriched with eggs flour is because eggs protein has higher digestibility in the human gastrointestinal tract, contains more EAAs and greater available AAs than plant protein. Plant proteins have lower digestibility than animal proteins, such as meat, poultry, egg, and milk [54]. Plants generally lack one or two NEAAs and are considered as low protein nutritional value source [55]. Lysine is the primary limiting AA in cereal proteins, whereas methionine and cysteine are in pulse proteins. The problem of AA deficiency can be overcome through the incorporation of protein sources, such as a mixture of cereals and legumes, plant, and animal proteins [56].

### 3.7. Texture Profile of Sago Noodle

This study used TPA and firmness methods to analyze the textural properties of noodles. TPA simulates the chewing process by compressing food samples twice to mimic the first and second bites, allowing for the measurement of several texture parameters [57]. Firmness testing focuses on the force required to shear or compress the sample, which often represents the most relevant characteristic in determining noodle quality [58, 59]. The TPA reveals that all sago noodle formulations were statistically different from one another across all evaluated textural parameters, as presented in Table 6.

**Table 6:** The noodles′ texture profile of sago starch (*n* = 6).

Parameters	Formulation		
	100% sago starch	80% sago starch and 20% egg powder	90% sago starch and 10% egg white powder
Hardness (g)	702.900 ± 149.258^b^	656.861 ± 81.455^c^	1080, 716 ± 59.130^a^
Adhesiveness (g.sec)	−15.041 ± 3.466^b^	−9.813 ± 3.290^a^	−13.869 ± 1,831^c^
Springiness	0.836 ± 0.015^a^	0.726 ± 0.05 ^c^	0.807 ± 0.028^b^
Cohesiveness	0.822 ± 0.024^a^	0.643 ± 0.022^c^	0.704 ± 0.017^b^
Gumminess	575.966 ± 115.247^b^	421.213 ± 40.519^c^	760.354 ± 45.100^a^
Chewiness	482.843 ± 104.323^b^	305.765 ± 28.133^c^	612.756 ± 31.850^a^
Resilience	0.623 ± 0.009^a^	0.427 ± 0.023^c^	0.525 ± 0.018^b^
Firmness (g)	513.322 ± 43.106^a^	137.136 ± 41.603^c^	189.474 ± 13.371^b^
Work of shear (g.sec)	276.058 ± 23.575^a^	98.841 ± 32.288 ^c^	142.327 ± 5.948 ^b^

*Note:* Values are means ± standard error, means followed by different letters in the same lines indicate the significant difference by the LSD test.

Hardness in TPA is the peak force required to compress the sample, reflecting its resistance to deformation [60]. Egg white powder fortification results in the highest hardness value, suggesting that egg white powder, rich in proteins (especially albumin), creates a denser, firmer network in the starch matrix. The proteins promote coagulation during cooking, reinforcing the noodle structure [61]. This is consistent with findings in noodle and pasta research where protein enrichment, particularly from albumin‐rich sources, increases hardness due to enhanced protein–starch interactions and network formation during heating and retrogradation. Such a protein–starch network has been reported to yield higher mechanical strength in gluten‐free and protein‐enriched noodle systems [62, 63].

Whole egg fortification gives a significantly lower hardness value compared to the egg white fortification, likely due to the fat content in the egg yolk, which interferes with the starch‐protein bonding, softening the structure [64]. This finding suggests that yolk lipids may interfere with the formation of a continuous matrix, acting as plasticizers that reduce rigidity. Similar effects have been noted in studies where lipid content modulates noodle texture by disrupting protein starch gel networks [65]. Without protein fortification, sago noodles have the lowest hardness due to the absence of protein contributions. The noodles rely solely on gelatinized sago starch for structure, which is essentially softer [66].

#### 3.7.1. Adhesiveness

Adhesiveness measures the stickiness of the sample, quantified as the negative force required to pull the probe away from the sample [67]. Egg‐white fortified noodles have the highest adhesiveness, whereas whole egg powder gives the opposite results, with the lowest adhesiveness. Reduced adhesiveness in egg‐fortified noodles reflects decreased surface stickiness due to protein and lipid interactions that limit free starch leaching and surface lubrication during compression. This aligns with studies showing that enhanced protein content can limit surface stickiness in noodle systems, potentially improving lubricity and processing performance [68].

Moreover, Lambrecht et al. [69] and Guo et al. [61] elaborated that egg‐white proteins interact with water to form sticky films, especially on the noodle surface, whereas the fat in the yolk may reduce stickiness by limiting water retention and preventing excessive hydration. Nonfortified sago noodles have moderate adhesiveness compared to the fortified ones due to the high water‐holding capacity of gelatinized sago starch, which contributes to stickiness but lacks the reinforcing effect of proteins [66].

#### 3.7.2. Springiness

Springiness is the extent to which a deformed sample returns to its original shape after compression [60]. Whole sago noodles have the significant highest springiness followed by egg‐white fortified, and the lowest in whole egg‐fortified noodles. This trend indicates that egg yolk components may weaken elastic recovery due to lipid interference with the starch matrix, consistent with findings in noodle texture studies where nonprotein constituents reduce elasticity [63].

The moderate springiness shown by egg‐white fortified noodles suggests that the coagulated proteins form a slightly elastic network, but the firmness of the structure limits full recovery. The highest springiness shown by non‐fortified sago noodles indicates that the gelatinized starch forms a more flexible matrix capable of recovering its shape after deformation. High springiness is often linked to a well‐developed gel network capable of reversible deformation, as discussed in recent noodle quality literature [70]. These results are also in line with the explanation presented by Lambrecht et al. [69] that the hydrophobic patches of proteins in whole egg and egg yolk noodles can interact with egg yolk lipids and thereby reduce the frequency of the occurrence of hydrophobic interactions between proteins.

#### 3.7.3. Cohesiveness

Cohesiveness measures the internal bonding strength of the sample and its ability to withstand compression without breaking [71]. The whole sago noodles have the significantly highest cohesiveness, followed by sago noodles with egg‐white powder and sago noodles with whole‐egg powder.

Lower cohesiveness with egg fortification suggests that added protein and lipid phases interrupt the uniform starch network, similar to reported effects when composite flours or additives interrupt continuous structural matrices in noodle products [70]. It is attributed to the fat in the yolk, which weakens the bonds between starch and protein [64]. Noodles with egg white fortification have moderate cohesiveness, demonstrating strong internal bonding due to protein cross‐linking, but not as much as pure sago noodles, which have the highest cohesiveness that reflect the uniform bonding of the gelatinized starch, without protein interference [65]. Egg white proteins, while increasing hardness and chewiness, still yield moderate cohesiveness, likely due to their ability to participate in network formation without excessive disruption from lipids.

#### 3.7.4. Gumminess

Gumminess is a measure of the energy required to disintegrate a sample, calculated as the product of hardness and cohesiveness [71]. Gumminess is highest for the 90% sago + 10% egg‐white powder noodles and lowest for 80% sago + 20% egg powder noodles. Increased gumminess in the egg‐white treatment aligns with the pronounced hardness and moderate cohesiveness, indicating a dense, robust structure due to protein‐starch interactions that provides higher resistance to deformation and a more compact matrix. Similar increases in gumminess have been reported in enriched noodles when added ingredients strengthen structural networks, such as in high‐protein or high‐dietary‐fiber systems [72].

The pure sago noodles demonstrate the lower gumminess, which is expected due to the lack of protein, making the noodles less resistant to chewing, as explained by Purwani et al. [73] and Sabbatini et al. [74]. The whole egg powder‐fortified noodles have the lowest gumminess due to the softening effect of yolk fat on the matrix [64].

#### 3.7.5. Chewiness

Chewiness measures the effort required to chew the sample, calculated as gumminess multiplied by springiness [60]. The egg‐white powder formulation demonstrates the highest chewiness, followed by the whole sago and whole‐egg powder noodles. The highest chewiness shown by egg‐white fortification aligns with its high gumminess and moderate springiness, making these noodles the most effortful to chew [75]. It suggests a more integrated and resilient matrix capable of sustaining repeated deformations, consistent with literature noting that increased protein integration enhances chewiness in noodle structures [63].

Moderate chewiness shown by whole sago noodles results from the balance of high springiness but lower gumminess [76]. Whole‐egg fortification leads to the lowest chewiness, suggesting the combined effects of softer texture and less springiness, as reported in the study of Anggraeni et al. [77].

#### 3.7.6. Resilience

Resilience measures how well the sample recovers energy after compression [76]. The egg‐white powder fortified noodles have moderate resilience, indicating the protein network partially absorbs compression energy but limits full recovery. Whole egg powder fortification results in noodles with lower resilience, showing that the presence of fat in starch reduces recovery capability. The results are in line with those of Mi et al. [78], who reported that the addition of protein led to the decline of noodles′ resilience. This decline suggests diminished ability to return to original shape due to interference with starch network elasticity, which has been found in similar studies where added fats reduce elastic recovery in starch‐based systems [70]. The highest resilience found in pure sago noodles can be explained by the elastic nature of pure gelatinized sago starch [66].

#### 3.7.7. Firmness

Firmness, representing the maximum force required to shear or compress the sample, demonstrated differences among the formulations [59]. Egg white‐fortified noodles exhibited a moderate firmness, reflecting the strengthening effect of coagulated proteins forming a robust network. Whole egg‐fortified noodles showed notably lower firmness, likely due to the plasticizing effect of yolk fat [61, 69]. In contrast, whole sago noodles displayed the highest firmness, attributed to the absence of protein that otherwise softens the starch matrix. This anomaly suggests that sago starch alone forms a rigid, gelatinized structure when unmodified by proteins or fats [66].

Harder formulations typically show higher firmness, but the relatively low values for fortified groups suggest that although egg proteins influence firmness, the plasticizing presence of yolk lipids in whole‐egg powder fortified noodles significantly reduces resistance to penetration, consistent with studies showing that added lipid phases decrease firmness in composite dough systems [63]. Egg‐white powder improves firmness compared to whole‐egg powder but does not reach the level shown by whole sago noodles, suggesting a trade off between protein network reinforcement and lipid weakening.

#### 3.7.8. Work of Shear

Work of shear quantifies the total mechanical energy needed to cut through or deform the noodle sample fully. The whole sago starch noodles required the most work of shear, highlighting the substantial energy needed to deform their rigid starch‐based network. This study aligns well with discussions on the significant energy required to deform sago‐based noodles due to their rigid starch network, which influences their textural properties [79]. Egg white powder fortified noodles required a high work of shear, although significantly lower compared to the whole sago noodles, consistent with their firm and cohesive texture. Research indicates that adding egg white powder to noodle formulations enhances protein aggregation through disulfide bond cross‐linking, leading to a higher work of shear than non‐fortified noodles [61, 80].

Whole egg powder fortified noodles required the least energy, corresponding to their softer and less cohesive texture. Whole egg powder fortification has been reported to impact noodle texture, contributing to lower work of shear due to the lipid content from egg yolk interfering with protein network development [70]. The results indicate that the whole sago noodles, although less hard than egg‐white powder fortified noodles, possess a more ductile and cohesive structure that resists total deformation, consistent with a highly interconnected starch network. Lower work of shear in fortified noodles highlights the structural fragmentation introduced by protein lipid interactions.

### 3.8. Color

Higher brightness (L∗) values indicate brighter color from the three sago noodle formulations, and the L∗ value is relatively the same as the moderate brightness level (Table 7). When compared to the L∗ value in the form of starch (Table 3), there is a decrease in the brightness level (L∗ value). The processing process causes a heating and gelatinization process, which changes the starch structure so that the color of the resulting product becomes darker and duller. The heating process unfolds the protein chain, allowing active groups (hydrophobic and electrostatic) to interact and form cross‐links, thereby creating a stable gel network [31, 81]. The formation of this gel influences the brightness level of the noodles.

**Table 7:** Results of measuring the color of noodle products.

Color	100% sago starch	80% sago starch and 20% whole egg powder	90% sago starch and 10% white egg powder
L∗	58.26 ± 2.21^a^	60.35 ± 2.38^a^	55.19 ± 3.36 ^a^
a∗	−1.43 ± 0.02^c^	1.42 ± 0.08^a^	0.18 ± 0.01^b^
b∗	7.50 ± 1.08^b^	15.27 ± 2.27^a^	8.25 ± 1.03^b^
C∗	7.61 ± 1.28^b^	15.35 ± 1.04^a^	8.28 ± 1.12^b^
Hue	100.78 ± 3.31^a^	84.88 ± 3.37 ^b^	89.06 ± 3.35^b^

*Note:* Values are means ± standard error, means followed by different letters in the same lines indicate the significant difference by the LSD test.

Meanwhile, noodles fortified with whole egg powder or egg white powder will experience a Maillard reaction due to the interaction between the amine groups in the protein and sugar, producing a brown compound that will reduce the brightness value. The heating process unfolds the protein chain, allowing active groups (hydrophobic and electrostatic) to interact and form cross‐links, thereby creating a stable gel network [31, 81]. The formation of this gel influences the brightness level of the noodles.

The (a∗) values of the three formulations showed significant differences, and the negative (a∗) value in the pure sago starch formulation showed a tendency towards green, although the intensity was not too dominant. The change from negative to positive (a∗) values (Table 7) showed a shift towards red, which most likely came from the egg yolk in whole egg powder. The positive (b∗) value indicated the presence of yellow nuances, but relatively low. A significant increase in the (b∗) value in the sago starch formulation with whole egg powder indicated a strong yellow nuance, resulting in a richer and more typical egg yolk color.

The value (C∗) indicates that the resulting color is less saturated (moderate saturation). The Hue value approaching 101° shows that the color tends to be between green and yellow, perhaps approaching the yellowish green. An almost two‐fold increase in the value (C∗) in the sago starch formulation with egg flour compared to the sago starch formulation indicates a much more saturated and intense color. The lower hue angle indicates a shift closer to yellow due to the contribution of egg yolk. According to Kusuma et al. [82], egg white powder has a high protein content, leading to a reaction between the amine group and glucose, which triggers the Maillard reaction. Based on the C∗ value of 8.28 and a Hue of 89.06, the color reflects a neutral yellow hue with moderate intensity, indicating the effect of egg white powder, which lacks significant pigment.

Adding whole egg powder to sago starch significantly increased brightness and color intensity (saturation) while shifting the hue towards reddish yellow. This effect is most likely due to the natural pigments in the egg yolk. Adding egg white powder had a different influence compared with whole egg powder. Because egg white flour lacks the yellow pigments found in egg yolk, although there was a slight increase in the (b∗) value, its overall effect on brightness and saturation was less pronounced. As a result, the noodles were not as bright or intense as those made with whole egg flour. However, the color of noodles can be improved by adding food coloring.

### 3.9. Scanning Electron Microscope

**Figure 3:**
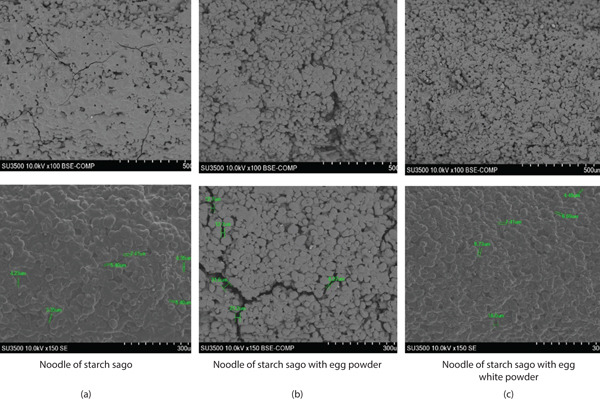
The microstructure in the cross‐section of the sago noodle. (a) Noodle of starch sago. (b) Noodle of starch sago with egg powder. (c) Noodle of starch sago with egg white powder.

The surface morphology of sago starch noodles (Figure 3a) appears uneven, with numerous pores and small particles scattered across the surface. It suggests that sago noodles have a porous structure, which may influence their mechanical properties and texture. The uneven surface could be caused by the starch components in sago, where starch gelatinization during the noodle‐making process contributes to this structural formation. Small cracks may indicate structural weakness caused by thermal or mechanical treatment. The presence of particles of varying sizes, ranging from approximately 3.35 to 6.35 *μ*m, may impact the texture and density of the noodles. The small pore in the image suggests that sago noodles have a porous structure, potentially enhancing water absorption during cooking. This characteristic also influences the noodles′ ability to bind sauces or liquids.

The surface morphology of noodles starch sago with egg powder (Figure 3b) appears more compact than sago noodles without added egg powder (Figure 3a). Several large cracks are visible in the image. These cracks may be caused by the brittle mixture of starch and egg protein during drying or processing. The particle size varies from 12 to 31.6 *μ*m, larger than sago noodles without added egg powder, indicating that the egg powder can affect the particle structure. The effect of the protein in the egg may act as a binder between starch particles, which causes aggregation and produces a more compact surface. The addition of eggs can also increase the mechanical strength of the noodle, but large cracks indicate areas of weakness due to uneven distribution. Morphological changes compared to the previous SEM image (Figure 3a) show differences in particle structure, with a more solid surface and fewer pores. The large cracks in the image indicate significant mechanical or thermal stress during the noodle‐making process, such as drying or rapid cooling. These cracks can affect the durability and texture quality of the noodles when cooked, such as breaking easily or losing elasticity.

The surface morphology of sago noodles with egg white flour (Figure 3c) showed a finer and denser granular structure. Adding egg white powder rich in albumin appeared to help fill the gaps between particles, resulting in a more compact structure. The particle size varied but appeared more uniform. Albumin can act as a tightening agent, resulting in a smoother surface. Cracks appeared fewer or no longer visible, indicating increased cohesion due to the binding effect of albumin protein. Albumin is an air‐soluble protein that has strong binding properties. In sago noodles, albumin can interact with starch particles, forming a denser and more elastic structure. The addition of albumin also reduced porosity, creating a smoother and more solid surface. With the presence of albumin protein, sago noodles tend to have a more elastic and chewy texture after cooking. This also helps prevent damage during processing or drying. With a more compact surface and minimal pores, this product is likely to be more resistant to mechanical damage during storage. This structure can also affect air absorption, making the noodles more stable when rehydrated. The addition of albumin provides the advantage of a smoother, chewier, and more break‐resistant texture, making it more attractive for culinary applications.

### 3.10. Implications for Process Optimization

Based on the TPA, whole egg fortification results in a softer texture with lower stickiness and moderate chewiness. In other terms, the noodles are easier to bite but less elastic. Egg white powder fortification results in firmer and chewier noodles, making them more elastic and resilient, which is desirable in many noodle products [70, 83]. Nonfortified noodles have a softer and springier texture but lack the firmness and chewiness desired for a robust noodle structure [78, 84].

The firmness testing shows that the noodles fortified with whole egg powder are soft and easy to shear, suitable for consumers who prefer tender noodles. Adding egg white powder increases the structural rigidity of the noodles, making them firmer and requiring more energy to shear [64]. Nonfortified noodles are unexpectedly the firmest, likely due to the absence of proteins that interact with starch to soften the structure [66].

The presented study showed that protein, especially egg white, contributes to enhanced hardness, cohesiveness, gumminess, and chewiness by forming a reinforced network with starch [78]. Egg yolk reduces these properties due to the plasticizing effect of its fat content [82]. Meanwhile, sago starch provides a highly cohesive and springy base matrix. However, it lacks the firmness and chewiness introduced by protein fortification [85].

Egg white powder is rich in albumin, and its proteins coagulate upon heating, forming a strong gel‐like network that binds with starch molecules. This results in increased hardness, gumminess, and chewiness. The stickiness (adhesiveness) may occur due to water retention at the noodle surface during cooking, as albumins can form hydrated gels [80]. Whole egg powder contains albumin (from egg white) and lipoproteins (from yolk). The lipids in the yolk interfere with protein–starch interactions, softening the noodle matrix and reducing hardness, gumminess, and chewiness. It makes whole egg‐fortified noodles suitable for consumers preferring tender textures [86].

Sago starch, primarily composed of amylose and amylopectin, undergoes gelatinization during cooking, creating a cohesive and elastic structure [85]. A secondary network within the gelatinized starch is formed with protein addition, enhancing firmness and chewiness (as seen with egg white). Fat in whole eggs disrupts this network, yielding softer noodles [84]. Based on the results above, egg white powder fortification is ideal for producing noodles with high elasticity and chewiness suitable for premium or artisanal noodle markets. Whole egg fortification works better for softer‐textured noodles, which are appealing in soups or for younger consumers [86]. Optimizing the starch‐to‐protein ratio can tailor noodles for specific applications. For instance, higher protein levels may be desirable for stir‐fried dishes, whereas lower levels suit soups. The control sample offers a springy texture, appealing to niche markets focused on traditional or gluten‐free products [85].

Stickiness and cohesiveness affect noodle handling during manufacturing. The higher adhesiveness in egg white‐fortified noodles may pose challenges in automated production lines, requiring adjustments in hydration or flour‐to‐protein ratios. Adjusting the hydration and protein levels can control stickiness, improving processability and consumer acceptability [81, 85]. Blending whole egg and egg white in varying proportions can create custom textures while managing costs [86]. Using additives such as emulsifiers (e.g., lecithin) may improve cohesiveness and reduce stickiness [83]. Extrusion parameters (e.g., moisture content, cooking time) can be fine‐tuned to control adhesiveness and resilience [85].

## 4. Conclusions

Fortification of sago starch with whole egg powder and egg white powder successfully increased protein content and improved the quality of the resulting noodles. A significant increase in protein was observed in formulations containing 80% sago starch and 20% whole egg powder (8.20% protein) and 90% sago starch and 10% egg white powder (8.64% protein). Additionally, changes in the pasting properties showed a decrease in PV, TV, and FV, whereas the reduction in BV indicated improved structural stability during heating. Fortification with egg white powder produced the best results in increasing compactness and reducing porosity and cracks on the noodle surface. These results demonstrate that fortification with egg powder is a promising approach for improving the quality of sago‐based food products, particularly in noodle applications.

## Author Contributions

Agus Budiyanto contributed to conceptualization, methodology, data curation, investigation, visualization, writing—original draft, and writing—review and editing. Risfaheri Risfaheri contributed to conceptualization, funding acquisition, methodology, investigation, visualization, supervision, writing—original draft, and writing—review and editing. Christina Winarti contributed to conceptualization, methodology, investigation, data curation, writing—original draft, and writing—review and editing. Widaningrum Widaningrum contributed to conceptualization, formal analysis, methodology, investigation, visualization, writing—original draft, and writing—review and editing. Sari Intan Kailaku contributed to conceptualization, methodology, investigation, visualization, software, writing—original draft, and writing—review and editing. Anna Sulistyaningrum contributed to conceptualization, methodology, investigation, visualization, writing—original draft, and writing—review and editing. Irpan Badrul Jamal contributed to investigation, data curation, resources, and writing—original draft. Tatang Hidayat contributed to data curation, visualization, validation, formal analysis, software, and writing—original draft. Agung Hendriadi contributed to data curation, formal analysis, visualization, validation, and writing—original draft. Abdullah Bin Arif contributed to conceptualization, methodology, data curation, investigation, visualization, writing—original draft, and writing—review and editing.

## Funding

No funding was received for this manuscript.

## Conflicts of Interest

The authors declare no conflicts of interest.

## Data Availability

The data that support the findings of this study are available from the corresponding author upon reasonable request.
